# Influence of *Cytochrome P450 2C19* Genotype on *Helicobacter pylori* Proton Pump Inhibitor-Amoxicillin-Clarithromycin Eradication Therapy: A Meta-Analysis

**DOI:** 10.3389/fphar.2021.759249

**Published:** 2021-10-15

**Authors:** Yuko Morino, Mitsushige Sugimoto, Naoyoshi Nagata, Ryota Niikiura, Eri Iwata, Mariko Hamada, Yusuke Kawai, Tatsuhiro Fujimiya, Hironori Takeuchi, Sakae Unezaki, Takashi Kawai

**Affiliations:** ^1^ Tokyo University of Pharmacy and Life Sciences, Tokyo, Japan; ^2^ Department of Gastroenterological Endoscopy, Tokyo Medical University Hospital, Tokyo, Japan; ^3^ Department of Practical Pharmacy, School of Pharmacy, Tokyo University of Pharmacy and Life Sciences, Tokyo, Japan; ^4^ Department of Pharmacy, Tokyo Medical University Hospital, Tokyo, Japan

**Keywords:** *H. pylori*, eradication, CYP2C19, tailored treatment, esomeprazole, rebaprezole

## Abstract

**Background:** Proton pump inhibitors (PPIs) are the first-line treatment for acid-related diseases. The pharmacokinetics and therapeutic efficacy of PPIs, however, are influenced by genetic factors such as variants in genes encoding drug-metabolizing enzymes (e.g., cytochrome P450 2C19 [CYP2C19]) and drug transporters. We performed a meta-analysis to evaluate the influence of CYP2C19 genotype and PPI class, PPI dose, treatment duration and clarithromycin dose on the cure rate of PPI-containing *Helicobacter pylori* eradication therapy.

**Methods:** Randomized control trials (RCTs) investigating cure rates using a PPI-amoxicillin-clarithromycin regimen among different CYP2C19 genotypes through May 2021 were included.

**Results:** A total of 25 studies (5,318 patients) were included. The overall eradication rate in the intention-to-treat analysis was 79.0% (3,689/4,669, 95% confidence interval [CI]: 77.8–80.2%), and that in CYP2C19 extensive metabolizers (EMs), intermediate metabolizer (IMs) and poor metabolizers (PMs) was 77.7% (1,137/1,464, 95% CI: 75.3–79.6%), 81.2% (1,498/1,844, 95% CI: 79.3–83.0%) and 86.8% (644/742, 95% CI: 83.9–88.9%), respectively. Meta-analysis showed that the relaTakashitive risk of failed eradication in CYP2C19 EMs compared with IMs and PMs was 1.21 (95% CI: 1.06–1.39, *P* = 0.006) and 1.57 (95% CI: 1.27–1.94, *P* < 0.001), respectively, in the fixed-effects model. The cure rate of omeprazole and lansoprazole-containing eradication regimens differed among CYP2C19 genotypes (*P* < 0.05), while that of rabeprazole and esomeprazole-containing regimens was similar.

**Conclusion:** The cure rates of PPI-amoxicillin-clarithromycin *H. pylori* eradication regimen, especially those containing omeprazole and lansoprazole, differ among CYP2C19 genotypes. Therefore, selection of a second-generation PPI or tailored treatment may achieve higher eradication rates than first-generation PPI-amoxicillin-clarithromycin triple regimen.

## Introduction

The Maastricht V/Florence Consensus Report issued by the European *Helicobacter* Study Group in 2017 provides a guideline on how to manage *Helicobacter pylori* (*H. pylori*) infection (Malfertheiner et al., 2017). This guideline indicates that, despite increasing *H. pylori* resistance rates to antibiotics such as clarithromycin (CAM), metronidazole (MNZ) and levofloxacin (LVFX) in most parts of the world, clinicians worldwide continue to use general first-line *H. pylori* triple eradication therapy consisting of a proton pump inhibitor (PPI) and two kinds of antimicrobial agents [i.e., CAM, amoxicillin (AMPC), MNZ, or LVFX], especially in areas with lower rates of the clarithromycin-resistant strain (Malfertheiner et al., 2017). In Japan, *H. pylori* eradication therapy is limited to regimens comprising an acid-inhibitory drug such as a PPI (e.g., esomeprazole, rabeprazole, lansoprazole and omeprazole) or vonoprazan at a standard dose at twice-daily dosing (bid), AMPC 750 mg bid, and CAM 200 mg or 400 mg bid for 7 days as a first-line regimen (Kato et al., 2019). This is despite the fact that eradication therapy for all *H. pylori*-positive patients with gastritis confirmed by endoscopy is currently covered by the Japanese National Health Insurance system. From 1990 to 2000, eradication rates achieved in Japan using PPI-AMPC-CAM triple therapy ranged from approximately 85–91% (Asaka et al., 2001). However, because this rate has subsequently decreased to 60–75%, the factors affecting cure rates of PPI-AMPC-CAM therapy need to be identified.

The cure rate for *H. pylori* infection may be affected by several factors, including antibiotic susceptibility (e.g., CAM, AMPC, MNZ and LVFX) (Asaka et al., 2001; Furuta et al., 2001b; Murakami et al., 2002), insufficient acid inhibition during eradication therapy (e.g., CYP2C19 and CYP3A4/5 genotype, PPI dose, treatment schedule and type of acid-inhibitory drug) (Furuta et al., 2001b; Sugimoto et al., 2007; Sugimoto and Yamaoka, 2009), poor adherence to medication, the environment (e.g., smoking), and the presence of an *H. pylori* strain with low virulence activity (e.g., *cagA*-negative strains, *vacA* s2 genotype and *dupA*-negative strains) (Sugimoto and Yamaoka, 2009; Shiota et al., 2012). Of these, acid inhibition for 24 h has re-emerged as an important factor for successful eradication therapy (Furuta et al., 2001b; Sugimoto et al., 2007; Sugimoto and Yamaoka, 2009). In fact, the European guideline states that “the use of high dose PPI bid increases the efficacy of triple therapy” (Malfertheiner et al., 2017).

PPIs are currently used as the first-line treatment for acid-related diseases. However, because PPIs undergo extensive hepatic metabolism by the cytochrome P450 (CYP) system, which includes CYP2C19 and CYP3A4 (Ishizaki and Horai, 1999), CYP2C19 genetic polymorphisms influence both the pharmacokinetics and pharmacodynamics (i.e., intragastric pH) of PPIs (Chang et al., 1995a; Chang et al., 1995b; Kubota et al., 1996; Ishizaki and Horai, 1999). At least 20 CYP2C19 variants have been identified, with the majority of patients with these variants being classified into three genotypes: extensive metabolizers (EMs), intermediate metabolizers (IMs), and poor metabolizers (PMs). The acid inhibition achieved using PPIs is greater in PMs than IMs or EMs because of the different pharmacokinetics among the three genotypes. Therefore, CYP2C19 polymorphisms are expected to influence the eradication rates of PPI-based eradication therapy. Many randomized control trials (RCTs) have investigated the efficacy of PPI-AMPC-CAM regimen among CYP2C19 genotypes (Dojo et al., 2001; Inaba et al., 2002; Isomoto et al., 2003; Kawabata et al., 2003; Miki et al., 2003; Take et al., 2003; Kuwayama et al., 2005; Okudaira et al., 2005; Sheu et al., 2005; Higuchi et al., 2006; Furuta et al., 2007; Hagiwara et al., 2007; Kuwayama et al., 2007; Lee et al., 2010; Zhang et al., 2010; Prasertpetmanee et al., 2013; Yang et al., 2015; Liou et al., 2016; Phiphatpatthamaamphan et al., 2016; Chunlertlith et al., 2017; Shimoyama et al., 2017; Ozaki et al., 2018; Arevalo Galvis et al., 2019; Chen et al., 2020; Lee et al., 2020). A meta-analysis conducted in 2013 to examine the effects in CYP2C19 PMs revealed significant differences in eradication rates between EMs and IMs (odds ratio (OR) 0.72; 95% confidence interval (CI): 0.59–0.88), between EMs and PMs (0.51; 0.38–0.68), and between IMs and PMs (0.69; 0.52–0.92) (Tang et al., 2013). However, this meta-analysis examined a small number studies (*n* = 16) that were written in English and Chinese and evaluated eradication by both PPI-AMPC-CAM and non-PPI-AMPC-CAM regimen (Tang et al., 2013). In addition, the year-by-year changes in the incidence rates of antimicrobial resistance mandate that the efficacy of these regimens be periodically re-evaluated.

Here, we performed a meta-analysis to re-evaluate the efficacy and safety of PPI-AMPC-CAM therapy alone among different CYP2C19 genotypes in RCTs written in English.

## Material Methods

### Search Strategy and Inclusion Criteria

This meta-analysis was conducted using data from RCTs identified by searching the medical literature on PubMed and Cochrane Library databases. We compared *H. pylori* eradication rates of PPI-AMPC-CAM triple therapy (each given over 7–14 days) as first-line therapy among different CYP2C19 genotypes (EMs, IMs and PMs). Two researchers (YM and MS) independently searched both the PubMed and Cochrane Library databases using the terms “*Helicobacter pylori,*” “eradication,” and “CYP2C19” and reviewed the titles and abstracts of all potential studies (Supplementary Figure S1). The inclusion criteria were 1) RCTs published through June 2021; 2) studies that used PPI-AMPC-CAM as first-line treatment; 3) studies comparing cure rates of PPI-AMPC-CAM regimen for *H. pylori* infection; and 4) studies written in English. Exclusion criteria were 1) non-RCT studies, 2) studies performed on non-PPI-AMPC-CAM regimen, 3) studies written in non-English languages, and 4) studies with abstracts alone. The author’s names, year of publication, country where the study was conducted, number of patients, eradication rate of each regimen, patient characteristics (sex and age), CYP2C19 genotype, susceptibility to antimicrobial agents, and incidence of adverse events were extracted from each study.

### Statistical Analysis

All meta-analyses were conducted using open-source statistical software (Review Manager Version 5.3. Copenhagen: The Nordic Cochrane Centre, The Cochrane Collaboration, 2014). First, a meta-analysis of RCTs comparing the cure rates of PPI-AMPC-CAM therapy in all patients and among *CYP2C19* genotypes was performed. For each comparison, intention-to-treat (ITT) and per-protocol (PP) analyses of cure rates were conducted. Relative risk (RR) and the corresponding 95% CI were used to summarize the effect of each comparison in fixed-effects models and random-effects models (46–48). Potential bias in each study was evaluated using funnel plots. Heterogeneity was evaluated using the *I* ^*2*^ value and Cochran’s Q. The *I* ^*2*^ values used to define heterogeneity were as follows: 0–39%, low heterogeneity; and 40–74%, moderate heterogeneity. All *p* values were two-sided, and *p* < 0.05 was considered statistically significant. Calculations were performed using commercial software (SPSS version 27, IBM Inc.; Armonk NY, United States).

## Results

### Literature Search and Data Extraction

The search strategy yielded 222 potentially eligible studies from PubMed and Cochrane Library databases and 15 studies from handsearching through other sources and papers (Supplementary Figure S1). A total of 120 were selected from the extracted studies. Of these, 44 studies involved non-PPI-AMPC-CAM regimen, seven investigated its use as second-line treatment, 22 studies were non-RCTs, 11 were reviews or meta-analyses, and 11 were written in a non-English language and were excluded. Ultimately, a total of 25 full articles were assessed for eligibility (Supplementary Figure S1) (Dojo et al., 2001; Inaba et al., 2002; Isomoto et al., 2003; Kawabata et al., 2003; Miki et al., 2003; Take et al., 2003; Kuwayama et al., 2005; Okudaira et al., 2005; Sheu et al., 2005; Higuchi et al., 2006; Furuta et al., 2007; Hagiwara et al., 2007; Kuwayama et al., 2007; Lee et al., 2010; Zhang et al., 2010; Prasertpetmanee et al., 2013; Yang et al., 2015; Liou et al., 2016; Phiphatpatthamaamphan et al., 2016; Chunlertlith et al., 2017; Shimoyama et al., 2017; Ozaki et al., 2018; Arevalo Galvis et al., 2019; Chen et al., 2020; Lee et al., 2020) and a total of 5,318 patients treated with a PPI-AMPC-CAM regimen for *H. pylori* infection were included in the analysis. Most of the studies (96%, 24/25) were performed in East and South-East Asian countries.

The characteristics of the trials investigating cure rates among *CYP2C19* genotypes are shown in Table 1. Eradication therapy comprised a regimen of a PPI (omeprazole (20 mg, bid), lansoprazole (30 or 60 mg, bid), rabeprazole (10 or 20 mg, bid), esomeprazole (20 or 40 mg, bid), ilaprazole (10 mg, bid) or dexlansoprazole (60 mg, once-daily dosing (oid), AMPC (750 or 1,000 mg, bid, or 500 mg, three-times-daily dosing (tid) or four-times-daily dosing (qid) and CAM (200, 400, or 500 mg, bid, 200 mg, tid or 1,000 mg, oid). Although most of the studies used an administration period of 7 days, one used 10 days (Arevalo Galvis et al., 2019), three used 14 days (Liou et al., 2016; Phiphatpatthamaamphan et al., 2016; Chen et al., 2020), one used 7–10 days (Lee et al., 2020) and one used 7–14 days (Prasertpetmanee et al., 2013) (Table 1).

**TABLE 1:** Characteristics of studies that investigated the *H. pylori* eradication outcome among CYP2C19 genotypes.

Authors	Year	Country	Number of patients	Age (mean)	Sex (M/F)	CYP2C19 EM (ITT/PP)	CYP2C19 IM (ITT/PP)	CYP2C19 PM (ITT/PP)	PPI	Regimen of PAC therapy (dose a day)	Duration
Dojo et al. Dojo et al. (2001)	2001	Japan	170	NA	83/81	NA/51	NA/77	NA/36	OPZ (20)/RPZ (20)	CAM (400) BID/AMPC (750) BID	7 days
Inaba et al. Inaba et al. (2002)	2002	Japan	183	NA	142/41	NA/65	NA/87	NA/27	OPZ (20)/LPZ (30)/RPZ (10)	CAM (200) TID/AMPC (500) TID	7 days
Kawabata et al. Kawabata et al. (2003)	2003	Japan	187	51.8 (20–78)	138 ⁄ 49	NA/63	NA/88	NA/22	LPZ (30)/RPZ (10)	CAM (400) BID/AMPC (750) BID	7 days
Miki et al. Miki et al. (2003)	2003	Japan	145	NA	105/33	NA/44	NA/72	NA/22	LPZ (30)/RPZ (10, 20)	CAM (400) BID/AMPC (1,000) BID	7 days
Take et al. Take et al. (2003)	2003	Japan	249	NA	219/30	81/72	125/119	43/40	OPZ (20)/LPZ (30)/RPZ (10)	CAM (400) BID/AMPC (750) BID	7 days
Isomoto et al. Isomoto et al. (2003)	2003	Japan	61	51.8 (21–73)	43/18	21/NA	28/NA	12/NA	LPZ (30)	CAM (200) BID/AMPC (750) BID	7 days
Sheu et al. Sheu et al. (2005)	2005	China	200	NA	99/101	91/83	65/60	44/42	OPZ (20)/EPZ (40)	CAM (500) BID/AMPC (1,000) BID	7 days
Okudaira et al. Okudaira et al. (2005)	2005	Japan	89	43.8 ± 1.0	89/0	NA/35	NA/46	NA/6	LPZ (30)	CAM (200) BID/AMPC (750) BID	7 days
Kuwayama et al. Kuwayama et al. (2005)	2005	Japan	225	52.7 ± 12.2	173/52	67/NA	119/NA	39/NA	OPZ (20)	CAM (400/500) BID/AMPC (750/1,000) BID	7 days
Higuchi et al. Higuchi et al. (2006)	2006	Japan	288	NA	176/112	82/NA	136/NA	67/NA	OPZ (20)	CAM (200/400) BID/AMPC (750) BID	7 days
Furuta et al. Furuta et al. (2007)	2007	Japan	150	60 (17–89)	100/50	52/NA	74/NA	24/NA	LPZ (30)	CAM (400) BID/AMPC (750) BID	7 days
Kuwayama et al. Kuwayama et al. (2007)	2007	Japan	479	NA	331/128	NA/149	NA/230	NA/80	RPZ (10, 20)	CAM (200/400) BID/AMPC (750) BID	7 days
Hagiwara et al. Hagiwara et al. (2007)	2007	Japan	22	50.6 (20–79)	14/6	NA/5	NA/8	NA/6	LPZ (30)	CAM (200) BID/AMPC (750) BID	7 days
Lee et al. Lee et al. (2010)	2010	Korea	492	NA	NA	NA/171	NA/219	NA/73	RPZ (20)/LPZ (30)	CAM (500) BID/AMPC (1,000) BID	7 days
Zhang et al. Zhang et al. (2010)	2010	China	240	NA	194/46	74/70	124/113	42/41	OPZ (20)/RPZ (10)	CAM (500) BID/AMPC (1,000) BID	7 days
Prasertpetmanee et al. Prasertpetmanee et al. (2013)	2013	Thailand	110	NA	39/71	NA/36	NA/19	NA/9	LPZ (60)	CAM (1,000) OID/AMPC (500) QID	7 days/14 days
Yang et al. Yang et al. (2015)	2015	Taiwan	150	54.3 ± 12.3	59/91	68/NA	63/NA	12/NA	RPZ (20)	CAM (500) BID/AMPC (1,000) BID	7 days
Liou et al. Liou et al. (2016)	2016	Taiwan	650	49.7 ± 12.9	328/322	NA/481^a^		NA/77	LPZ (30)	CAM (500) BID/AMPC (1,000) BID	14 days
Phiphatpatthamaamphan et al. Phiphatpatthamaamphan et al. (2016)	2016	Thailand	50	53.6 (29–70)	17/33	NA/25	NA/21	NA/2	RPZ (20)	CAM (1,000) OID/AMPC (500) QID	14 days
Chunlertlith et al. Chunlertlith et al. (2017)	2017	Thailand	170	NA	85/85	74/72	78/71	16/15	OPZ (20)	CAM (500) BID/AMPC (1,000) BID	7 days
Shimoyama et al. Shimoyama et al. (2017)	2017	Japan	200	NA	90/99	NA/60	NA/129^b^	NA/NA	EPZ (20/RPZ (10)	CAM (200) BID/AMPC (750) BID	7 days
Ozaki et al. Ozaki et al. (2018)	2018	Japan	147	NA	74/73	NA/19	NA/33	NA/9	EPZ (20)/RPZ (10)	CAM (200) BID/AMPC (750) BID	7 days
Arévalo Galvis et al. Arevalo Galvis et al., (2019)	2019	Colombia	69	45.7 ± 13	23/46	59/53	10/10	NA	OPZ (20)	CAM (500) BID/AMPC (1,000) BID	10 days
Chen et al. Chen et al. (2020)	2020	China	338	51.0 ± 13.3	175/163	NA/185^a^		NA/31	Dexlansoprazole (60) OID	CAM (500) BID/AMPC (1,000) BID	14 days
Lee et al. Lee et al. (2020)	2020	Korea	254	NA	151/103	NA/72	NA/122	NA/36	Ilaprazole (10)	CAM (500) BID/AMPC (1,000) BID	7 days/10 days

AMPC, amoxicillin; BID, twice-daily dosing; CAM, clarithromycin; D, day; EM, extensive metabolizer of CYP2C19; EPZ, esomeprazole; IM, intermediate metabolizer of CYP2C19; ITT, intention-to-treat; LPZ, lansoprazole; MNZ, metronidazole; NA, not available; OID, once-daily dosing; OPZ, omeprazole; PM, poor metabolizer of CYP2C19; PP, per-protocol; PPI, proton pump inhibitor; QID, four-times-daily dosing; RPZ, rabeprazole.

a Combined number of CYP2C19 EMs and IMs.

b Combined number of CYP2C19 IMs and PMs.

All studies genotyped patients for CYP2C19 variants. Two studies combined the number of CYP2C19 EMs and IMs (Liou et al., 2016; Chen et al., 2020) and one combined the number of IMs and PMs (Shimoyama et al., 2017). The prevalence of CYP2C19 EMs, IMs and PMs was 35.4% (1,357/3,834), 48.0% (1,844/3,838), and 16.6% (633/3,834), respectively.

### Meta-Analysis of Eradication Rate of PPI-AMPC-CAM Therapy

All 25 trials were analyzed for the efficacy of the eradication therapy used. The ITT and PP cure rates of the PPI-AMPC-CAM therapy were 79.0% (3,689/4,669, 95% CI 77.8–80.2%) and 84.0% (4,232/5,039, 95% CI 82.9–85.0%), respectively (Table 2). When we divided *H. pylori*-positive patients by CYP2C19 genotype, the cure rate was 77.7% (1,137/1,464, 95% CI: 75.3–79.6%) in CYP2C19 EMs, 81.2% (1,498/1,844, 95% CI: 79.3–83.0%) in IMs and 86.8% (644/742, 95% CI: 83.9–88.9%) in PMs (Table 2).

**TABLE 2:** Eradication rates of PPI-amoxicillin-clarithromycin triple therapy among CYP2C19 genotypes.

Authors	Number of patients	Eradication rate (ITT/PP)	CYP2C19 EM (ITT/PP)	CYP2C19 IM (ITT/PP)	CYP2C19 PM (ITT/PP)
Dojo et al. Dojo et al. (2001)	170	NA/81.7%	NA/76.5%	NA/84.4%	NA/86.1%
Inaba et al. Inaba et al. (2002)	183	82.0%/83.8%	Na/75.4%	NA/88.5%	NA/88.9%
Kawabata et al. Kawabata et al. (2003)	187	72.2%/78.0%	NA/79.4%	NA/78.4%	NA/72.7%
Miki et al. Miki et al. (2003)	145	82.8%/87.0%	Ma/88.6%	NA/88.9%	NA/77.3%
Take et al. Take et al. (2003)	249	74.3%/80.1%	77.8%/77.8%	74.4%/78.2%	83.7%/90.0%
Isomoto et al. Isomoto et al. (2003)	61	80.3%/84.5%	76.2%/NA	78.6%/NA	91.7%/NA
Sheu et al. Sheu et al. (2005)	200	82.5%/89.2%	76.9%/84.3%	94.6%/91.7%	90.9%/95.2%
Okudaira et al. Okudaira et al. (2005)	89	76.4%/78.2%	NA/62.9%	NA/87.0%	NA/100%
Kuwayama et al. Kuwayama et al. (2005)	225	80.9%/81.4%	80.6%/NA	79.8%/NA	84.6%/NA
Higuchi et al. Higuchi et al. (2006)	288	80.6%/82.9%	74.4%/NA	84.6%/NA	82.1%/NA
Furuta et al. Furuta et al. (2007)	150	70.0%/72.9%	57.7%/NA	71.6%/NA	91.7%/NA
Kuwayama et al. Kuwayama et al. (2007)	479	NA/89.1%	NA/85.9%	NA/88.7%	NA/96.3%
Hagiwara et al. Hagiwara et al. (2007)	22	72.7%/70.0%	NA/60.0%	NA/75.0%	NA/83.3%
Lee et al. Lee et al. (2010)	492	82.5%/75.2%	NA/71.3%	NA/76.7%	NA/79.5%
Zhang et al. Zhang et al. (2010)	240	82.5%/88.4%	82.4%/87.1%	79.8%/87.6%	90.5%/92.7%
Prasertpetmanee et al. Prasertpetmanee et al. (2013)	110	96.4%/96.4%	NA/94.4%	NA/100%	NA/100%
Yang et al. Yang et al. (2015)	150	80.7%/81.2%	76.4%/NA	85.7%/NA	78.9%/NA
Liou et al. Liou et al. (2016)	650	85.7%/91.0%	NA/84.8%	NA/NA	NA/94.8%
Phiphatpatthamaamphan et al. Phiphatpatthamaamphan et al. (2016)	50	86.0%/87.8%	NA/92.0%	NA/81.0%	NA/100%
Chunlertlith et al. Chunlertlith et al. (2017)	170	80.6%/89.5%	87.8%/90.3%	71.8%/78.9%	87.5%/93.3%
Shimoyama et al. Shimoyama et al. (2017)	200	73.0%/77.2%	NA/75.0%	NA/78.3%	NA/NA
Ozaki et al. Ozaki et al. (2018)	147	72.8%/72.8%	NA/63.2%	NA/78.8%	NA/77.8%
Arévalo Galvis et al. Arevalo Galvis et al. (2019)	69	84.1%/92.1%	83.1%/92.5%	90.0%/90.0%	NA/NA
Chen et al. Chen et al. (2020)	338	84.3%/88.0%	NA/89.2%	NA/NA	NA/93.6%
Lee et al. Lee et al. (2020)	254	70.1%/76.4%	NA/79.2%	NA/75.4%	NA/72.2%

EM, extensive metabolizer of CYP2C19; IM, intermediate metabolizer of CYP2C19; ITT, intention-to-treat; NA, not available; PM, poor metabolizer of CYP2C19; PP, per-protocol.

**FIGURE 1:**
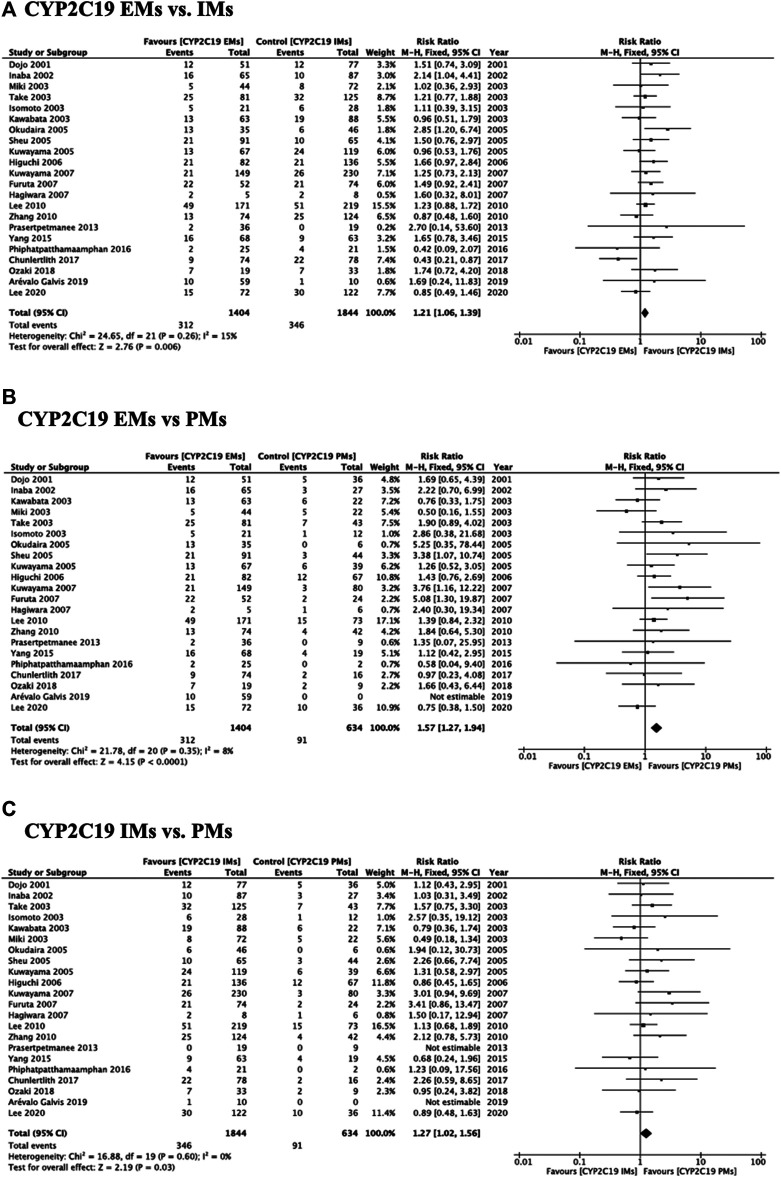
Forest plots of *H. pylori* cure rates of PPI-amoxicillin-clarithromycin regimen among different *CYP2C19* genotypes in the fixed-effects model. **(A)** *CYP2C19* EMs vs IMs, **(B)** EMs vs PMs and **(C)** IMs and PMs.

Meta-analysis showed that the RR of failed eradication in CYP2C19 EMs compared with IMs and PMs was 1.21 (95% CI: 1.06–1.39, *p* = 0.006) and 1.57 (95% CI: 1.27–1.94, *p* < 0.001) in the fixed-effects model (Figures 1A,B (95% CI: 1.05–1.43, *p* = 0.010) and 1.44 (95% CI: 1.14–1.81, *p* = 0.002) in the random-effects model (Supplementary Figure S2A and Supplementary S2B), respectively. The fixed-effects model also showed that there was a significant increase in RR in CYP2C19 IMs compared with PMs (1.27, 95% CI: 1.02–1.56, *p* = 0.030) (Figure 1C). There was low heterogeneity among the studies (*CYP2C19* EMs vs IMs: Chi^2^ = 24.65, *p* = 0.26, I^2^ = 15%; EMs vs PMs: Chi^2^ = 21.78, *p* = 0.35, I^2^ = 8%; and IMs vs PMs: Chi^2^ = 16.88, *p* = 0.60, I^2^ = 0%) (Figure 1). The funnel plots of all included studies showed symmetry between different genotypes (Supplementary Figure S3).

### Subgroup Analysis of Eradication Rate by Class of PPI

We divided patients based on their use of four PPIs: omeprazole (Dojo et al., 2001; Inaba et al., 2002; Kuwayama et al., 2005; Sheu et al., 2005; Higuchi et al., 2006; Zhang et al., 2010; Chunlertlith et al., 2017; Arevalo Galvis et al., 2019), lansoprazole (Inaba et al., 2002; Isomoto et al., 2003; Kawabata et al., 2003; Miki et al., 2003; Okudaira et al., 2005; Furuta et al., 2007; Hagiwara et al., 2007; Lee et al., 2010; Prasertpetmanee et al., 2013; Liou et al., 2016), rabeprazole (Dojo et al., 2001; Inaba et al., 2002; Kawabata et al., 2003; Miki et al., 2003; Kuwayama et al., 2007; Lee et al., 2010; Zhang et al., 2010; Yang et al., 2015; Phiphatpatthamaamphan et al., 2016; Ozaki et al., 2018; Lee et al., 2020) and esomeprazole (Sheu et al., 2005; Shimoyama et al., 2017; Ozaki et al., 2018) (Table 3). The ITT and PP cure rates of omeprazole-AMPC-CAM therapy were 81.3% (891/1,095, 95% CI: 78.9–83.6%) and 86.1% (961/1,116, 95% CI: 83.9–88.1%), respectively (Table 3). The ITT cure rates of lansoprazole, rabeprazole and esomeprazole were 80.9% (1,235/1,515, 95% CI: 79.5–83.4%), 81.1% (1,485/1,831, 95% CI: 79.2–82.9%) and 80.0% (216/270, 95% CI: 74.7–84.6%), respectively, which were similar among different PPI-containing treatment regimens (*p* = 0.998) (Table 3).

**TABLE 3:** Studies that investigated *H. pylori* eradication outcome among CYP2C19 genotypes.

Authors	PPI	Number of patients (ITT/PP)	CYP2C19 EM (ITT/PP)	CYP2C19 IM (ITT/PP)	CYP2C19 PM (ITT/PP)	Eradication rate (ITT/PP)	CYP2C19 EM (ITT/PP)	CYP2C19 IM (ITT/PP)	CYP2C19 PM (ITT/PP)
Dojo et al. Dojo et al. (2001)	OPZ (20)	NA/86	NA/30	NA/36	NA/20	NA/81.4%	NA/73.3%	NA/86.1%	NA/85.0%
Inaba et al. Inaba et al. (2002)	OPZ (20)	59/58	NA/21	NA/27	NA/10	83.1%/84.5%	NA/76.2%	NA/88.9%	NA/90.0%
Sheu et al. Sheu et al. (2005)	OPZ (20)	100/93	45/41	32/30	23/22	79.0%/84.9%	68.9%/75.6%	84.4%/90.0%	91.3%/95.5%
Kuwayama et al. Kuwayama et al. (2005)	OPZ (20)	225/215	67/NA	119/NA	39/NA	80.9%/84.7%	80.6%/NA	79.8%/NA	84.6%/NA
Higuchi et al. Higuchi et al. (2006)	OPZ (20)	288/280	82/NA	136/NA	67/NA	80.6%/82.6%	74.4%/NA	84.6%/NA	82.1%/NA
Zhang et al. Zhang et al. (2010)	OPZ (20)	120/109	39/36	61/54	20/19	79.2%/87.2%	71.8%/77.8%	80.3%/90.7%	89.0%/94.7%
Chunlertlith et al. Chunlertlith et al. (2017)	OPZ (20)	170/153	74/72	78/71	16/15	80.6%/89.5%	87.8%/90.3%	71.8%/78.9%	87.5%/93.3%
Arévalo Galvis et al. Arevalo Galvis et al. (2019)	OPZ (20)	133/122	111/101	21/20	1/1	88.0%/95.9%	87.4%/96.0%	90.5%/95.5%	100%/100%
Inaba et al. Inaba et al. (2002)	LPZ (30)	60/58	NA/20	NA/29	NA/9	86.7%/89.7%	NA/90.0%	NA/89.7%	NA/88.9%
Miki et al. Miki et al. (2003)	LPZ (30)	49/47	NA/12	NA/26	NA/9	79.6%/83.0%	NA/83.3%	NA/84.6%	NA/77.8%
Kawabata et al. Kawabata et al. (2003)	LPZ (30)	87/80	NA/33	NA/35	NA/10	69.0%/75.0%	NA/72.7%	NA/74.3%	NA/100%
Isomoto et al. Isomoto et al. (2003)	LPZ (30)	61/58	21/NA	28/NA	12/NA	80.3%/84.5%	76.2%/NA	78.6%/NA	91.7%/NA
Okudaira et al. Okudaira et al. (2005)	LPZ (30)	89/87	NA/35	NA/46	NA/6	76.4%/78.2%	NA/62.9%	NA/87.0%	NA/100%
Furuta et al. Furuta et al. (2007)	LPZ (30)	150/144	52/NA	74/NA	24/NA	70.0%/72.9%	57.7%/NA	71.6%/NA	91.7%/NA
Hagiwara et al. Hagiwara et al. (2007)	LPZ (30)	22/20	NA/5	NA/8	NA/6	63.6%/70.0%	NA/60.0%	NA/75.0%	NA/83.3%
Lee et al. Lee et al. (2010)	LPZ (30)	247/234	NA/85	NA/108	NA/41	74.9%/79.1%	NA/74.1%	NA/80.6%	NA/85.4%
Prasertpetmanee et al. Prasertpetmanee et al. (2013)	LPZ (60)	110/110	NA/36	NA/19	NA/9	96.4%/96.4%	NA/94.4%	NA/100%	NA/100%
Liou et al. Liou et al. (2016)	LPZ (30)	650/602	NA/481^a^		NA/77	85.7%/92.5%	NA/84.8%^a^		NA/94.8%
Dojo et al. Dojo et al. (2001)	RPZ (20)	NA/78	NA/21	NA/41	NA/16	NA/83.3%	NA/81.0%	NA/82.9%	NA/87.5%
Inaba et al. Inaba et al. (2002)	RPZ (10)	64/63	NA/24	NA/31	NA/8	76.6%/77.8%	NA/62.5%	NA/87.1%	NA/87.5%
Miki et al. Miki et al. (2003)	RPZ (10, 20)	96/91	NA/32	NA/46	NA/13	85.4%/90.1%	NA/90.6%	NA/91.3%	NA/76.9%
Kawabata et al. Kawabata et al. (2003)	RPZ (10)	100/93	NA/30	NA/53	NA/10	75.0%/80.6%	NA/86.7%	NA/81.1%	NA/60.0%
Kuwayama et al. Kuwayama et al. (2007)	RPZ (10, 20)	479/459	NA/149	NA/230	NA/80	85.4%/89.1%	NA/85.9%	NA/88.7%	NA/96.3%
Lee et al. Lee et al. (2010)	RPZ (20)	245/229	NA/86	NA/111	NA/32	66.5%/71.2%	NA/68.6%	NA/73.0%	NA/71.9%
Zhang et al. Zhang et al. (2010)	RPZ (10)	120/115	35/34	63/59	22/22	85.8%/89.6%	94.3%/97.1%	79.4%/84.7%	90.9%/90.9%
Yang et al. Yang et al. (2015)	RPZ (20)	450/445	199/NA	192/NA	57/NA	87.1%/88.1%	85.9%/NA	90.1%/NA	87.7%/NA
Phiphatpatthamaamphan et al. Phiphatpatthamaamphan et al. (2016)	RPZ (20)	100/97	NA/44	NA/47	NA/6	90.0%/92.8%	NA/95.4%	NA/93.6%	NA/83.3%
Ozaki et al. Ozaki et al., (2018)	RPZ (10)	76/76	NA/6	NA/19	NA/3	68.4%/68.4%	NA/66.7%	NA/73.7%	NA/100%
Lee et al. Lee et al. (2020)	RPZ (10)	101/95	NA/30	NA/65^b^		70.3%/74.8%	NA/76.7%	NA/73.8%^b^	
Sheu et al. Sheu et al. (2005)	EPZ (40)	100/92	46/42	33/30	21/20	86.0%/93.5%	84.8%/92.9%	84.8%/93.3%	90.5%/95.0%
Ozaki et al. Ozaki et al. (2018)	EPZ (20)	71/71	NA/13	NA/14	NA/6	77.5%/77.5%	NA/61.5%	NA/85.7%	NA/66.7%
Shimoyama et al. Shimoyama et al. (2017)	EPZ (20)	99/94	NA/30	NA/64^b^		75.8%/79.8%	NA/73.3%	NA/82.8%^b^	

EM, extensive metabolizer of CYP2C19; EPZ, esomeprazole; IM, intermediate metabolizer of CYP2C19; ITT, intention-to-treat; LPZ, lansoprazole; NA, not available; OPZ, omeprazole; PM, poor metabolizer of CYP2C19; PP, per-protocol; PPI, proton pump inhibitor; RPZ, rabeprazole.

a Combined number of CYP2C19 EMs and IMs.

b Combined number of CYP2C19 IMs and PMs.

The cure rates of omeprazole-, lansoprazole-, rabeprazole- and esomeprazole-AMPC-CAM therapy in CYP2C19 EMs were 79.4% (374/469), 73.6% (220/299), 83.4% (547/656) and 77.5% (69/89), respectively (*p* = 0.696). Those in CYP2C19 PMs were 85.7% (168/196), 91.6% (196/203), 87.0% (215/247) and 88.5% (23/26), respectively (*p* = 0.972).

**FIGURE 2:**
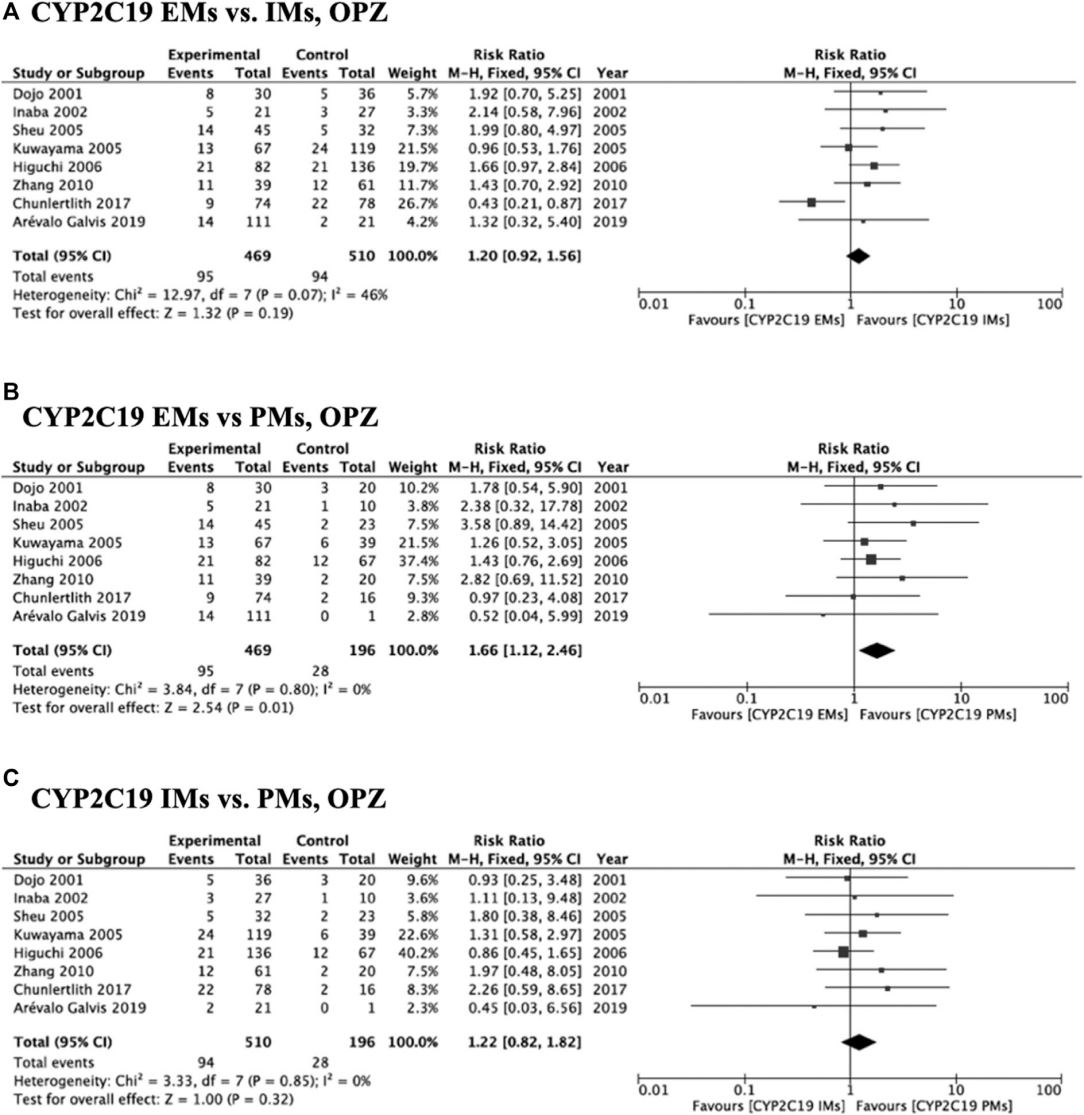
Forest plots of *H. pylori* cure rates of OPZ-amoxicillin-clarithromycin regimen among different *CYP2C19* genotypes in the fixed-effects model. **(A)** *CYP2C19* EMs vs IMs, **(B)** EMs vs PMs and **(C)** IMs and PMs.; Abbreviations: CI, confidence interval; EM, extensive metabolizer of CYP2C19; IM, intermediate metabolizer of CYP2C19; OPZ, omeprazole; PPI, proton pump inhibitor; PM, poor metabolizer of CYP2C19; PPI, proton pump inhibitor.

**FIGURE 3:**
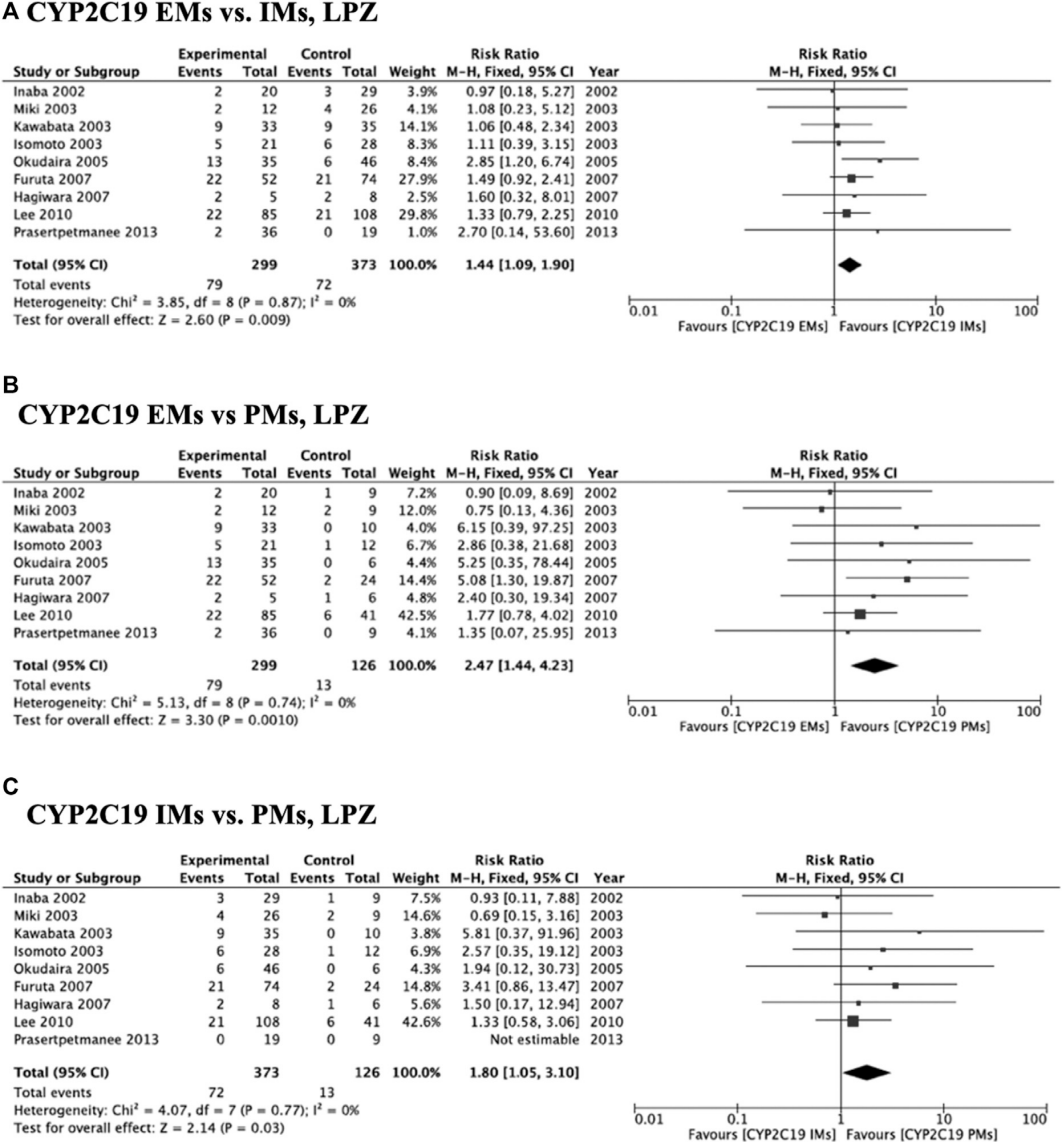
Forest plots of *H. pylori* cure rates of LPZ-amoxicillin-clarithromycin regimen among different *CYP2C19* genotypes in the fixed-effects model. **(A)** *CYP2C19* EMs vs IMs, **(B)** EMs vs PMs and **(C)** IMs and PMs.; Abbreviations: CI, confidence interval; EM, extensive metabolizer of CYP2C19; IM, intermediate metabolizer of CYP2C19; LPZ, lansoprazole; PPI, proton pump inhibitor; PM, poor metabolizer of CYP2C19; PPI, proton pump inhibitor.

**FIGURE 4:**
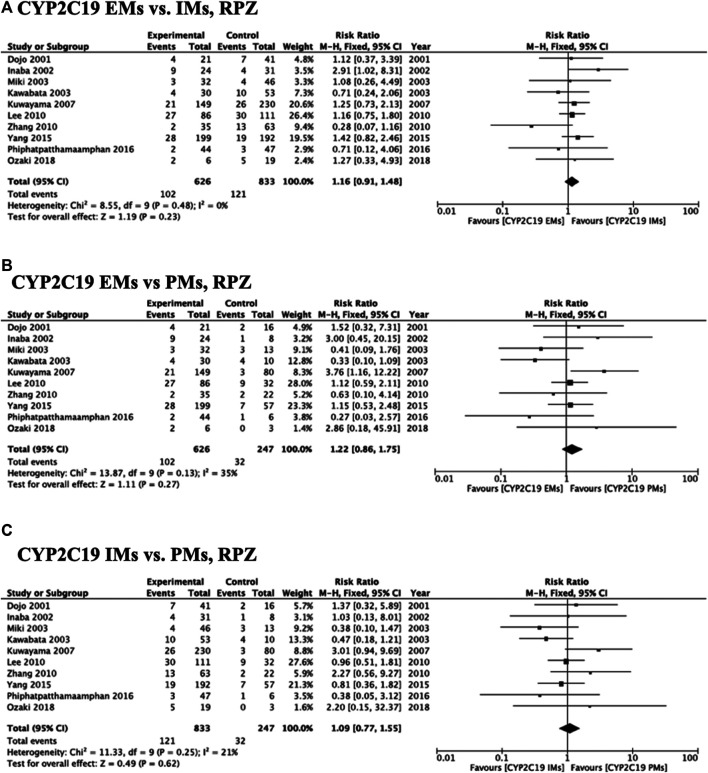
Forest plots of *H. pylori* cure rates of RPZ-amoxicillin-clarithromycin regimen among different *CYP2C19* genotypes in the fixed-effects model. **(A)** *CYP2C19* EMs vs IMs, **(B)** EMs vs PMs and **(C)** IMs and PMs.; Abbreviations: CI, confidence interval; EM, extensive metabolizer of CYP2C19; IM, intermediate metabolizer of CYP2C19; PPI, proton pump inhibitor; PM, poor metabolizer of CYP2C19; PPI, proton pump inhibitor; RPZ, rabeprazole.

**FIGURE 5:**
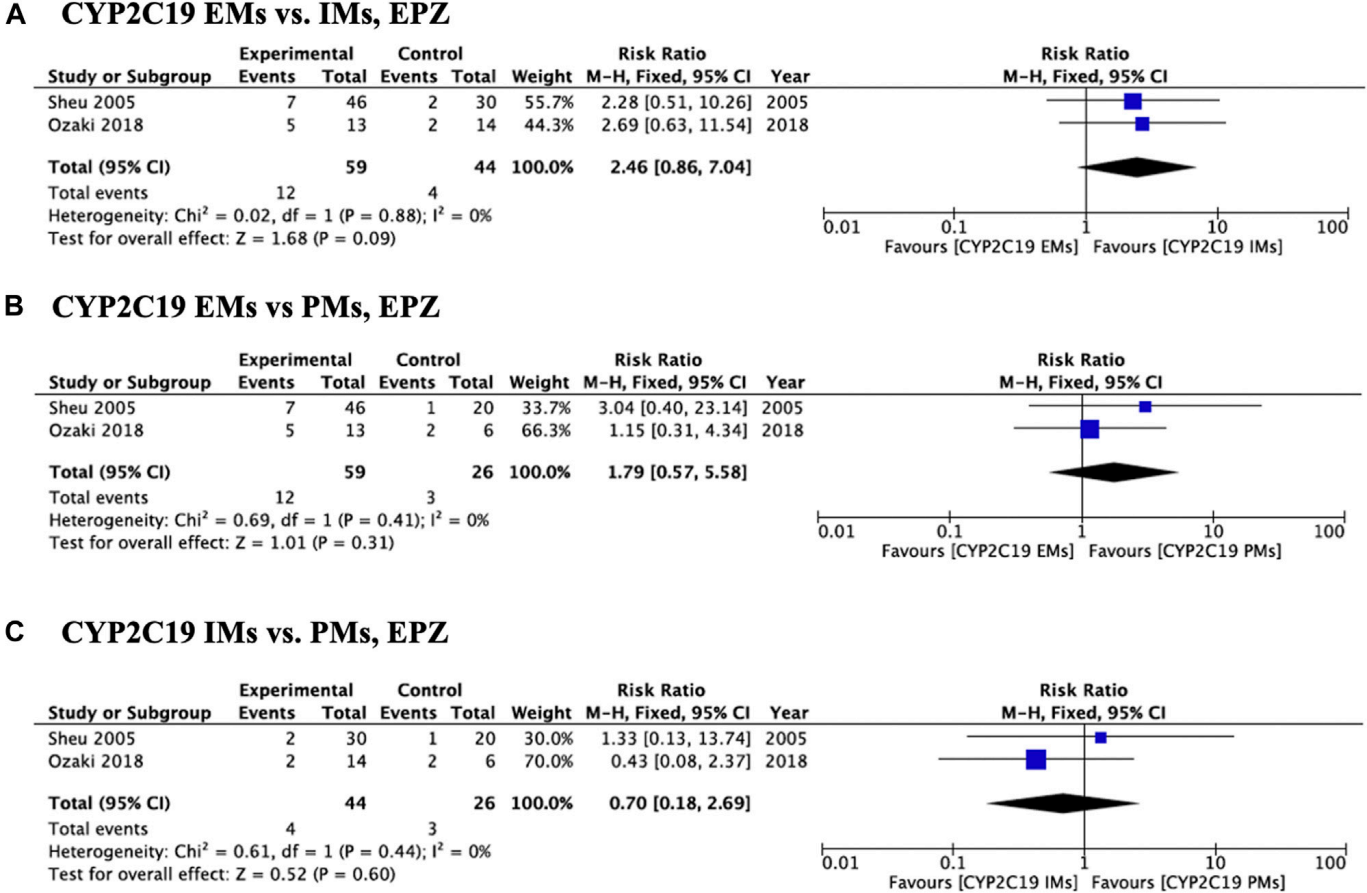
Forest plots of *H. pylori* cure rates of EPZ-amoxicillin-clarithromycin regimen among different *CYP2C19* genotypes in the fixed-effects model. **(A)** *CYP2C19* EMs vs IMs, **(B)** EMs vs PMs and **(C)** IMs and PMs.; Abbreviations: CI, confidence interval; EM, extensive metabolizer of CYP2C19; EPZ, esomeprazole; IM, intermediate metabolizer of CYP2C19; PPI, proton pump inhibitor; PM, poor metabolizer of CYP2C19; PPI, proton pump inhibitor.

Meta-analysis of studies that used omeprazole and lansoprazole as the first-generation PPI showed that the RR of failed eradication in CYP2C19 EMs compared with PMs was 1.66 (95% CI: 1.12–2.46, *p* = 0.01) and 2.47 (95% CI: 1.44–4.23, *p* = 0.001), respectively, in the fixed-effects model (Figure 2B, Figure 3B). In contrast, studies that used rabeprazole and esomeprazole showed no significant differences in the RR of failed eradication among the three genotypes (Figures 4, Figure 5). There was low to moderate heterogeneity among these studies (Figures 2–5).

### Subgroup Analysis of Eradication Rates Between CAM-Sensitive and CAM-Resistant Cases

Sixteen studies (68%, 17/25) reported data on susceptibility to clarithromycin (Inaba et al., 2002; Kawabata et al., 2003; Miki et al., 2003; Take et al., 2003; Kuwayama et al., 2005; Sheu et al., 2005; Higuchi et al., 2006; Furuta et al., 2007; Hagiwara et al., 2007; Kuwayama et al., 2007; Yang et al., 2015; Liou et al., 2016; Phiphatpatthamaamphan et al., 2016; Chunlertlith et al., 2017; Ozaki et al., 2018; Chen et al., 2020) (Supplementary Table S1). Resistance rates to CAM, AMPC and MNZ were 13.1% (372/2,840), 8.9% (109/1,227), and 29.9% (165/552), respectively. Cure rates in patients infected with CAM-sensitive and resistant strains were 89.7% (1787/1992, 95% CI: 88.3–91.0%) and 42.1% (142/337, 95% CI: 36.8–47.6%), respectively.

### Subgroup Analysis of Eradication Rates Among Different PPI Dose, Among Different Treatment Duration and Among Different CAM Dose

We divided studies based on their dose of PPIs: standard-dose (omeprazole: 20 mg, lansoprazole: 30 mg, rabeprazole: 10 mg and esomeprazole 20 mg) and double dose (omeprazole: 20 mg, lansoprazole: 60mg, rabeprazole: 20 mg and esomeprazole 40 mg). The cure rates of standard-dose-PPI therapy and double-dose-PPI therapy were 80.6% (1,847/2,292) and 85.2% (901/1,057), respectively (Table 4). Meta-analysis of studies that standard-dose-PPI therapy showed that the RR of failed eradication in CYP2C19 EMs compared with IMs and PMs was 1.28 (95% CI: 1.07–1.53) and 1.72 (95% CI: 1.30–2.29), respectively. In contrast, studies that used double-dose-PPI therapy showed no significant differences in the RR of eradication among the three CYP2C19 genotypes (Table 4).

**TABLE 4:** Effect of CYP2C19 genotypes on the eradication rate of PPI-based triple therapies among different dose of PPI, among different duration of eradication and different dose of CAM.

	Eradication rate	EM	IM	PM	EM vs IM	*p* value	EM vs PM	*p* value	IM vs PM	*p* value
	Total				95%CI		95%CI		95%CI	
**PPI-dose**										
Standard dose	80.6% (1847/2,292)	77.3% (664/859)	81.4% (866/1,064)	85.9% (317/369)	1.28 (1.07–1.53)	< 0.01	1.72 (1/30–2.29)	< 0.01	1.29 (0.97–1.71)	0.08
Double dose	85.2% (901/1,057)	83.8% (373/445)	86.5% (403/466)	85.6% (125/146)	1.31 (0.96–1.78)	0.09	1.19 (0.77–1.84)	0.43	0.90 (0.58–1.40)	0.65
**Treatment duration**										
7-days	80.3% (2,902/3,615)	76.8% (975/1,270)	81.0% (1,406/1736)	85.6% (521/609)	1.22 (1.06–1.41)	< 0.01	1.60 (1.29–1.98)	< 0.01	1.29 (1.04–1.61)	0.02
10 and 14 days	81.5% (349/428)	86.4% (127/147)	79.5% (175/220)	77.0% (47/61)	0.82 (0.52–1.29)	0.38	0.70 (0.39–1.26)	0.18	0.82	0.74
**CAM-dose**										
400 mg/day	83.1% (654/787)	76.2% (199/261)	85.6% (333/389)	89.1% (122/137)	1.62 (1.17–2.25)	< 0.01	2.15 (1.25–3.69)	< 0.01	1.39 (0.81–2.39)	0.23
800–1000 mg/day	80.1% (2,479/3,093)	78.4% (894/1,141)	80.1% (1,165/1,455)	84.5% (420/497)	1.12 (0.96–1.30)	0.15	1.46 (1.16–1.84)	< 0.01	1.23 (0.98–1.55)	0.77

CAM, clarithromycin; EM, extensive metabolizer of CYP2C19; IM, intermediate metabolizer of CYP2C19; PM, poor metabolizer of CYP2C19; PPI, proton pump inhibitor

We divided studies based on their treatment duration: 7 days and 10–14 days. The cure rates of 7-days therapy and 10–14 days therapy were 80.3% (2,902/3,615) and 81.5% (349/428), respectively (Table 4). Meta-analysis of studies that 7-days therapy showed that the RR of failed eradication in CYP2C19 EMs compared with IMs and PMs was 1.22 (95% CI: 1.06–1.41) and 1.72 (95% CI: 1.29–1.98), respectively. In contrast, studies that used 10–14 days therapy showed no significant differences in the RR of failed eradication among CYP2C19 genotypes.

The cure rates of CAM 400 mg/day therapy and 800–1,000 mg/day therapy were similar [83.1% (654/787) and 80.1% (2,479/3,093), respectively] (Table 4). Meta-analysis of studies that CAM 400 mg/day and 800–1,000 mg/day therapies showed that the RR of failed eradication in CYP2C19 EMs compared with PMs were 2.15 (95% CI: 1.25–3.69) and 1.46 (95% CI: 1.16–1.84), respectively.

### Incidence of Adverse Events due to PPI-AMPC-CAM Therapy

All studies reported data on adverse events, namely diarrhea, loose stools, dysgeusia, and abdominal pain (Supplementary Table S2). The incidence of adverse events due to PPI-AMPC-CAM therapy was 38.1% (1,175/3,084, 95% CI: 36.4–39.8%) (Supplementary Table S2).

## Discussion

This meta-analysis included 25 RCTs from 24 Asian and 1 South American country to re-evaluate the association between the cure rate of first-line PPI-AMPC-CAM triple *H. pylori* eradication therapy and CYP2C19 genotype. Of a total of 5,318 patients with resistance rates of 13.1% for CAM and 8.9% for AMPC, the overall cure rate in ITT analysis was 79.0% (95% CI: 77.8–80.2%). Meta-analysis indicated that cure rates significantly differed among CYP2C19 genotypes, and the RR of failed eradication in CYP2C19 EMs compared with IMs and PMs was 1.21 (95% CI: 1.06–1.39) and 1.57 (95% CI: 1.27–1.94), respectively, in the fixed-effects model. In addition, although sub-analysis of studies that used omeprazole and lansoprazole showed that the RR in CYP2C19 EMs compared with PMs was 1.66 (95% CI: 1.12–2.46) and 2.47 (95% CI: 1.44–4.23), there were no significant differences in RR in studies that used rabeprazole and esomeprazole among genotypes. These results suggest that when PPI-containing triple regimen is selected at the eradication therapy for *H. pylori* infection, second-generation PPI or high-dose of first-generation PPI should be used with triple therapy, especially in Asian populations.

### Acid Inhibition and *H. pylori* Eradication Therapy

An optimal intragastric pH during prolonged potent acid inhibition in the stomach increases the stability and bioavailability of acid-sensitive antibacterial agents (e.g., CAM and AMPC) (Grayson et al., 1989; Hunt, 1993). This in turn leads to an increase in the gastric mucosal concentration of antimicrobial agents and exhibits antibacterial effects (Grayson et al., 1989; Goddard et al., 1996; Scott et al., 1998). In addition, acid inhibition increases the susceptibility of *H. pylori* to antimicrobial agents (Scott et al., 1998). Therefore, rapid, potent and prolonged neutralization of pH after optimal PPI treatment is required to cure *H. pylori* infection and increases eradication rate. We previously showed, using a PPI/AMPC/CAM regimen, that the median 24-h pH during eradication therapy was higher (6.4) and the median pH < 4 holding time ratio (HTR) (0.5%) was shorter in patients who experienced successful eradication than those who experienced failed eradication (pH 5.2 and pH < 4 HTR 26.7%) (Sugimoto et al., 2007). Because the degree and duration of acid inhibition correlates with the cure rate, pH > 4 should be maintained for 24 h, and a target pH higher than 6.0 is needed for acid inhibition to cure patients using PPI/AMPC/CAM therapy, irrespective with infection of CAM-resistant strain.

A critical subsequent question then becomes: how do we plan treatment using acid inhibitory drugs to achieve this target pH for acid inhibition? Controlling pH with PPIs depends on the dosing schedule, dose, and a combination of acid inhibitors (Shirai et al., 2001; Shirai et al., 2002; Sugimoto et al., 2004; Sugimoto et al., 2005; Sugimoto et al., 2012). Further, polymorphisms in genes encoding drug-metabolizing enzymes and drug transporters such as *CYP2C19* and multidrug resistance protein-1 (*ABCB1*) affect pH during treatment (Furuta et al., 1999; Shirai et al., 2001; Shirai et al., 2002; Sugimoto et al., 2004; Sugimoto et al., 2005; Kodaira et al., 2009; Sugimoto et al., 2012). In general, the pharmacokinetics and pharmacodynamics of PPIs significantly differ among CYP2C1*9* genotypes. A previous meta-analysis reported significantly different cure rates of PPI therapy in patients with gastroesophageal reflux disease, a major acid-related disease, with different *CYP2C19* genotypes (ITT analysis: EMs, 52.2%; IMs, 56.7%; PMs, 61.3%; *p* = 0.047), and that CYP2C19 EMs had an increased risk of being refractory to PPI therapy compared to PMs (OR: 1.661, 95% CI 1.023–2.659, *p* = 0.040) (Ichikawa et al., 2016). Therefore, it is necessary to note that some patients who become resistant to PPI treatment may be CYP2C19 EMs. It may be possible to overcome this disadvantage of genetic variations affecting response to PPIs by using frequent dosing of PPIs, which has been shown to be more efficacious for acid inhibition through 24 h than a one-time increase in dose (Furuta et al., 2001c; Sugimoto et al., 2004; Lou et al., 2009). This phenomenon is due to the fact that frequent dosing sustains plasma levels of PPIs through a 24-h period and continues to inactivate H^+^, K^+^-ATPase consistently for 24 h (Furuta et al., 2001c; Sugimoto et al., 2004; Lou et al., 2009). However, because acid inhibition by omeprazole, lansoprazole, rabeprazole and esomeprazole bid in CYP2C19 EMs is 5.0 (2.4–5.9), 4.7 (3.7–5.5), 4.8 (2.5–6.4) and 5.4 (3.5–6.8) (Sahara et al., 2013), respectively, maintaining acid secretion for 24 h in all patients may be difficult. In fact, a previous meta-analysis conducted in 2013 showed that CYP2C19 EM is a risk factor for eradication (Tang et al., 2013). In the present study, we re-evaluated the effect of *CYP2C19* genotype on the cure rate of first-line PPI-AMPC-CAM eradication therapy by analyzing RCTs written in English and demonstrated that the cure rate was 77.7% in CYP2C19 EMs, 81.2% in IMs and 86.8% in PMs, and that the RR of failed eradication in CYP2C19 EMs compared with IMs and PMs was 1.21 and 1.57, respectively. Because the incidence rates of antimicrobial resistance change year-by-year, determining the antibiotic susceptibility of *H. pylori* using either culture or genetic testing or both is of great importance, particularly in populations with a high rate of drug-resistant strains. Therefore, we think our finding that CYP2C19 genotype has a major effect on patients’ response to first-line PPI-AMPC-CAM therapy has great clinical relevance.

In 2015, the potassium-competitive acid blocker, vonoprazan, became clinically available in Japan. Vonoprazan competitively inhibits H^+^/K^+^-ATPase activity more firstly and potently than PPIs (Kagami et al., 2016). Therefore, a vonoprazan-containing eradication regimen is expected to increase the eradication rate compared with PPI-containing conventional regimens. In fact, a recent meta-analysis that investigated the efficacy of first-line eradication therapy showed that a vonoprazan-containing regimen achieved a higher eradication rate than a PPI-containing regimen (88.1% (95%CI: 86.1–89.9%) in the vonoprazan therapy and 72.8% (95%CI: 71.0–75.4%) in PPI therapy) (Jung et al., 2017; Sugimoto and Yamaoka, 2018). Because eradication rate for patients infected with CAM-sensitive strain is similar between vonoprazan and PPI, a benefit by vonoprazan-induced potent acid inhibition may play in patients infected with CAM-resistance strain. Therefore, as the first-line standard treatment for *H. pylori* eradication in Japan, a vonoprazan-containing regimen is selected. However, there is no study to investigate the efficacy and safety of vonoprazan-AMPC-CAM therapy among CYP2C19 genotypes by the RCT. Further trials should be planned to clarify association with vonoprazan and CYP2C19 genotypes and association with PPI/vonoprazan and other genetic variation including CYP3A4 (Sugimoto et al., 2020).

### Class of PPI and *H. pylori* Eradication Therapy

PPIs can be divided into first generation (e.g., omeprazole and lansoprazole) and second generation (e.g., rabeprazole and esomeprazole), which differ in the degree to which they are metabolized by CYP2C19. Rabeprazole is metabolized to thioether-rabeprazole mainly *via* a non-enzymatic pathway, with minor contribution from CYP2C19 (Yasuda et al., 1995). Esomeprazole is a pure S-isomer of omeprazole and interindividual variations in plasma concentrations of the S-isomer are lower than those in the R-isomer. In this meta-analysis, we found that the cure rates of omeprazole-, lansoprazole-, rabeprazole- and esomeprazole-AMPC-CAM therapy were similar. However, for first-generation PPIs, the RR of failed eradication in CYP2C19 EMs compared with PMs was 1.66 (95% CI 1.12–2.46) for omeprazole and 2.47 (1.44–4.23) for lansoprazole. In contrast, there were no significant differences in cure rates of regimen using second-generation PPIs among genotypes. In addition, because second generation PPIs are more effective for eradication therapy than first generation PPIs, second-generation PPIs or vonoprazan should be selected to ensure high efficacy in all patients, irrespective of CYP2C19 genotype.

### CAM Resistance and *H. pylori* Eradication Therapy

There is growing evidence that the cure rate of PPI-AMPC-CAM regimen decreases with an increase in CAM-resistant strains, and recent rates of CAM-resistant strains in Japan and Europe have exceeded 35 and 20%, respectively (Furuta et al., 2001a; Asaka et al., 2001; Murakami et al., 2002; Megraud, 2007). Although previous studies have reported that cure rates in patients infected with CAM-resistant strains have markedly decreased to 20–40% (Fischbach and Evans, 2007; Megraud, 2007), this meta-analysis obtained cure rates of 89.7% (95% CI 88.3–91.0%) and 42.1% (95% CI 36.8–47.6%) in patients infected with CAM-sensitive and resistant strains, respectively. The Maastricht V/Florence Consensus Report recommends first-line therapy using a CAM-containing regimen with PPI/AMPC or PPI/MNZ and an alternative treatment using bismuth-containing quadruple treatment in areas with low prevalence of CAM-resistant strains or bismuth or non-bismuth quadruple treatment and concomitant therapies in areas with high (>15%) CAM resistance (Malfertheiner et al., 2017). This indicates the need to carefully select antimicrobial agents and/or regimens based on individual’s antibiotic resistance to *H. pylori* and/or known regional characteristics.

Recent studies have demonstrated the efficacy of tailored treatment based on sensitivity to CAM, with eradication rates exceeding 90% (Furuta et al., 2007; Kawai et al., 2008; Sugimoto et al., 2014). Although it is unfeasible to provide tailored treatment based on sensitivity to CAM to all patients with *H. pylori* infection or all areas/populations around the world due to limitations in culture testing and PCR in clinical practice, tailored treatment based on sensitivity to CAM should be considered in areas with a high incidence rate of CAM-resistant *H. pylori*.

Because eradication rate for patients infected with CAM-sensitive strain is similar between vonoprazan and PPI (Jung et al., 2017; Sugimoto and Yamaoka, 2018), insufficient acid inhibition by PPI in CYP2C19 EMs may reduce eradication rate for CAM-resistance *H. pylori* strain. Therefore, different efficacy among CYP2C19 genotypes shown by our meta-analysis may depends on rate of CAM-resistance strain in each study. This observation will support hypothesis that potent acid inhibition (e.g., PPI high-dose, PPI qid with high adherence, vonoprazan and tailored PPI therapy based on CYP2C19 genotype) is important to be high eradication rate in patients infected with CAM-resistant strain (Furuta et al., 2007; Sugimoto et al., 2014). In fact, despite the dual therapy of vonoprazan/AMPC, it was reported to show a high eradication rate in patients infected with CAM-resistant strain (Suzuki et al., 2020). Although this observation suggests that when vonoprazan and AMPC are selected for eradication therapy, CAM may be unnecessary. Therefore, we think that culture test and susceptibility-based treatment provides best outcome for *H. pylori* eradication therapy. Although potency of PPI differs among kind of PPIs and resistant rate to CYP2C19 differs among kind of PPIs, acid inhibition by PPI also depends on the dosing schedule (oid, bid and qid) and dose. Therefore, I should be evaluated to determine optimal eradication regimen by combined analysis with various factors (dosing schedule, dose, genetic factor, potency, and generation) that affect intragastric pH at the time of PPI administration, as further study.

### Limitations

This meta-analysis has a few limitations. First, there is a possibility of selection bias due to the exclusion of studies published in languages other than English, those with abstracts alone and studies that used non-RCT designs. Second, only one RCT has examined the association of CYP2C19 genotype with cure rate outside of East Asia. Third, the small sample size may have affected the statistical power of sub-group analyses. Because background (e.g., race and sex), treatment (e.g., class of PPI, regimen, dose of PPI, kinds of antimicrobial agents) and bacterial factors (e.g., resistance to antimicrobial agents and virulence factors) are associated with the outcome of eradication therapy, future meta-analyses of a larger number of RCTs should account for these factors to gain a clearer understanding of the association of cure rate with CYP2C19 genotype. Forth, because there were a few reports investigating effect of CYP2C19 genotype between clarithromycin-sensitive and resistant strain, we cannot meta-analyze associations in this time.

## Conclusion

Cure rates of first-line PPI-AMPC-CAM eradication therapy differ among CYP2C19 genotypes, especially in Asian populations. This meta-analysis suggest that PPI-AMPC-CAM therapy has the potential to eradicate *H. pylori* infection in >85% of CYP2C19 PMs, but a low proportion of CYP2C19 EMs. Thus, the cure rate of this regimen in CYP2C19 EMs may be insufficient to eradicate *H. pylori* infection. Therefore, genotyping patients for CYP2C19 variants before eradication therapy may help to achieve higher eradication rates. To receive eradication rate >90% is important in clinical practice (Graham et al., 2007). However, most of regimen cannot reach to this goal, due to insufficient acid inhibition during treatment and susceptibility to antimicrobial agents. Therefore, second generation PPI-high-dose, high-frequent (qid) or vonoprazan-containing tailored treatment based on susceptibility to antimicrobial agents may optimal to ignore CYP2C19 genotype status and to receive high eradication rate.

## Data Availability

The original contributions presented in the study are included in the article/Supplementary Material, further inquiries can be directed to the corresponding author.

## Abbreviations

CI, confidence interval; EM, extensive metabolizer of CYP2C19; IM, intermediate metabolizer of CYP2C19; PPI, proton pump inhibitor; PM, poor metabolizer of CYP2C19; PPI, proton pump inhibitor.
