# Supplementary figures and images for: Influence of Cytochrome P450 2C19 Genotype on Helicobacter pylori Proton Pump Inhibitor-Amoxicillin-Clarithromycin Eradication Therapy: A Meta-Analysis
Source: Front Pharmacol. 2021 Oct 15;12:759249. doi: 10.3389/fphar.2021.759249 (PMC8553963; doi:10.3389/fphar.2021.759249)

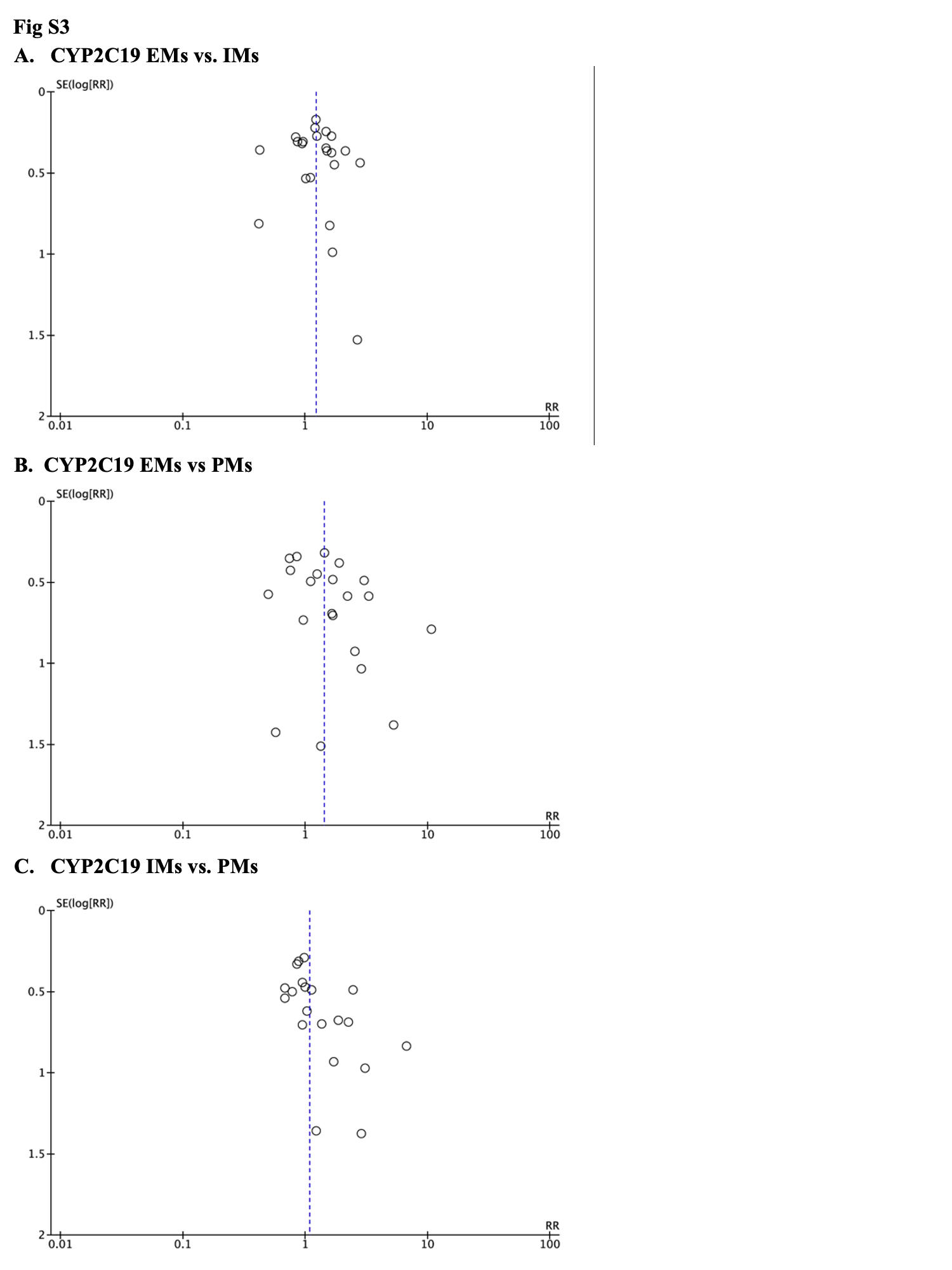

Supplement: Supplementary file 1 [file Image3.TIFF]

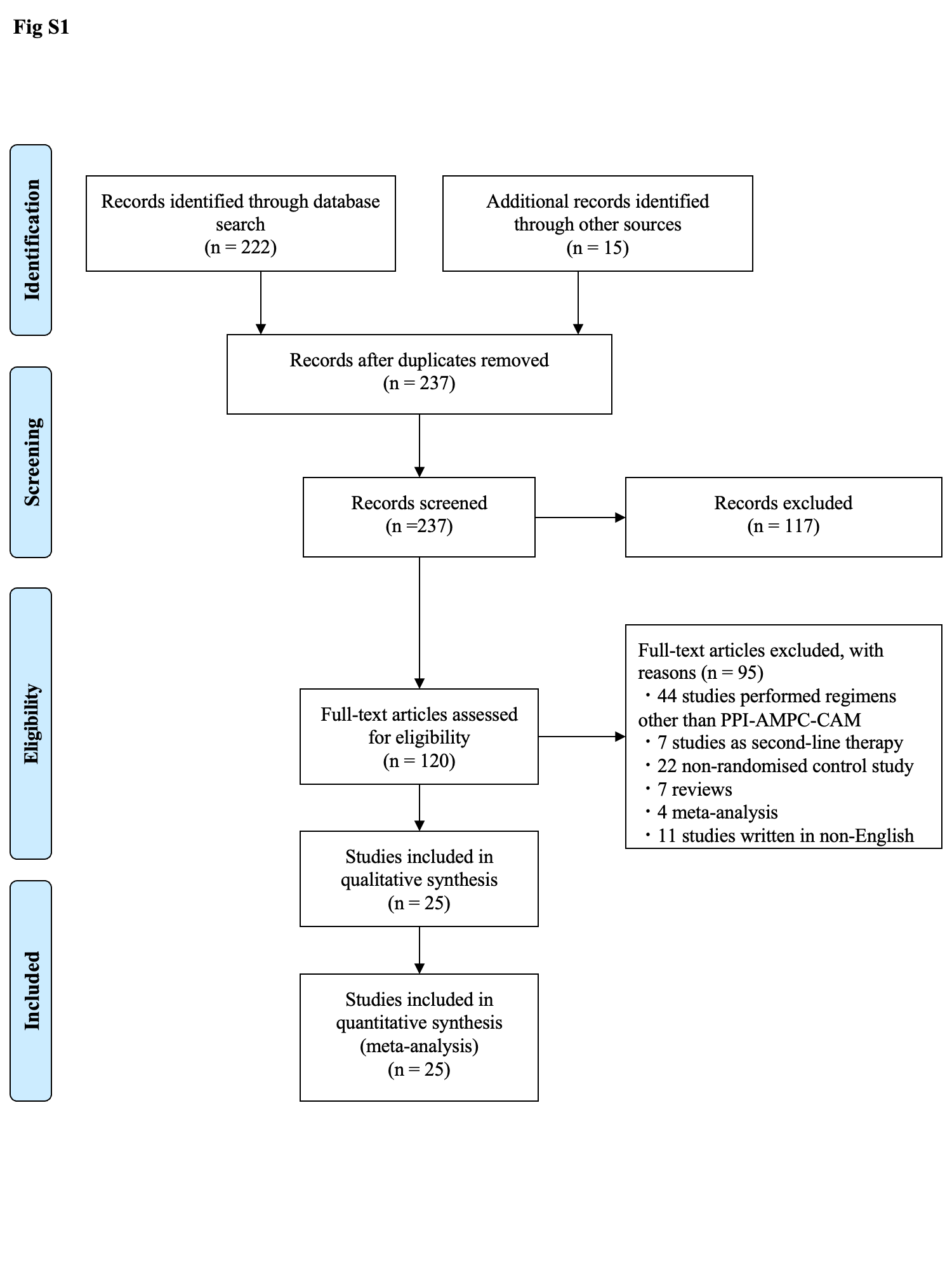

Supplement: Supplementary file 2 [file Image1.TIFF]

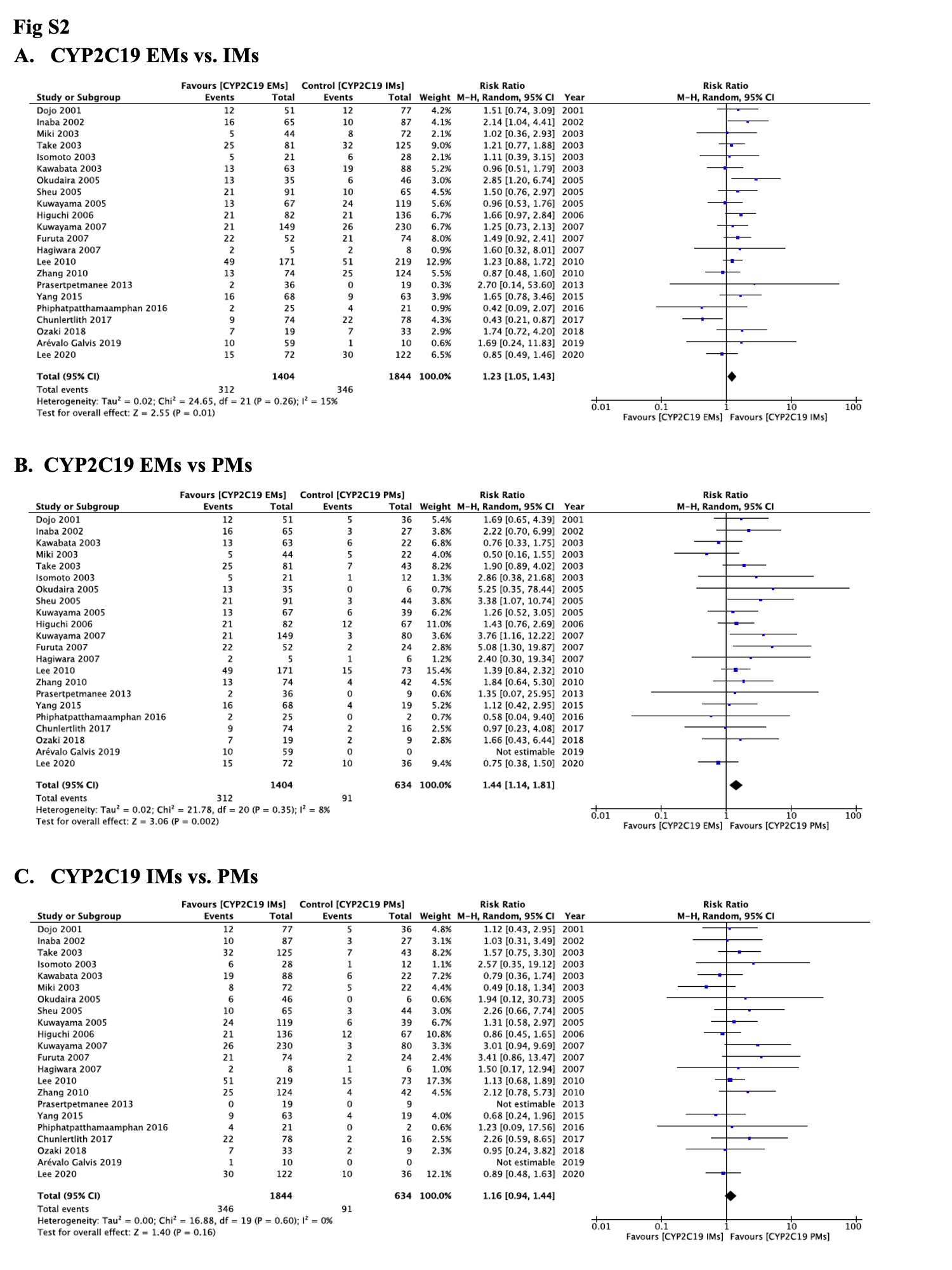

Supplement: Supplementary file 4 [file Image2.TIFF]
