# Supplementary material for: Influence of Cytochrome P450 2C19 Genotype on Helicobacter pylori Proton Pump Inhibitor-Amoxicillin-Clarithromycin Eradication Therapy: A Meta-Analysis
Source: Front Pharmacol. 2021 Oct 15;12:759249. doi: 10.3389/fphar.2021.759249 (PMC8553963; doi:10.3389/fphar.2021.759249)
Supplement: Supplementary file 3 [file Table1.docx]

**Table S1.** Resistance strains to antimicrobial agents and eradication rates of PPI-amoxicillin-clarithromycin triple therapy

| Authors | Number | Clarithromycin  n (resistance rate) | Amoxicillin  n (resistance rate) | Metronidazole  n (resistance rate) | Eradication rate  Clarithromycin-sensitive strain | Eradication rate  Clarithromycin-resistant strain | Eradication rate  Metronidazole-sensitive strain | Eradication rate  Metronidazole-resistant strain |
| --- | --- | --- | --- | --- | --- | --- | --- | --- |
| Inaba et al. (1) | 161 | 1 (0.6%) |  |  |  |  |  |  |
| Kawabata et al. (2) | 173 | 21 (12.1%) |  |  | 85.5% (130/152) | 23.8% (5/21) |  |  |
| Miki et al. (3) | 138 | 16 (11.6%) |  |  | 97.5% (119/122) | 6.3% (1/16) |  |  |
| Take et al. (4) | 249 | 17 (6.8%) |  |  | 77.6% (180/232) | 29.4% (5/17) |  |  |
| Isomoto et al. (5) | NA |  |  |  | 84.2% (48/57) | 25.0% (1/4) |  |  |
| Sheu et al. (6) | 139 | 10 (7.2%) |  |  |  |  |  |  |
| Kuwayama et al. (7) | 225 | 36 (16.0%) | 35 (15.6%) |  | 88.4% (167/189) | 41.7% (15/36) |  |  |
| Higuchi et al. (8) | 285 | 43 (15.1%) | 57 (20.0%) |  | 87.3% (213/244) | 41.9% (18/43) |  |  |
| Furuta et al. (9) | 150 | 44 (29.3%) |  |  | 81.1% (86/106) | 43.2% (19/44) |  |  |
| Kuwayama et al. (10) | 439 | 60 (13.7%) |  |  | 95.0% (360/379) | 50.0% (30/60) |  |  |
| Hagiwara et al. (11) | 16 | 1 (6.3%) |  |  |  |  |  |  |
| Yang et al. (12) | 150 | 26 (17.3%) | 1 (0.7%) | 50 (33.3%) | 93.5% (116/124) | 19.2% (5/26) | 83.0% (83/100) | 76.0% (38/50) |
| Liou et al. (13) | 402 | 36 (9.0%) | 3 (0.7%) | 115 (28.6%) | 91.5% (335/366) | 44.4% (16/36) | 89.5% (257/287) | 81.7% (94/115) |
| Phiphatpatthamaamphan et al. (14) | UN | 4 (-) |  | 9 (-) |  |  |  |  |
| Chunlertlith et al. (15) | 86 | 6 (7.0%) |  |  |  |  |  |  |
| Ozaki et al. (16) | 62 | 25 (40.3%) |  |  | 86.5% (32/37) | 48.0% (12/25) |  |  |
| Chen et al. (17) | 165 | 30 (18.2%) | 13 (7.9%) |  | 96.3% (131/136) | 66.7% (20/30) |  |  |

Abbreviations: NA, not available; UA, unknown

**Table S2.** Adverse events due to PPI-amoxicillin-clarithromycin triple therapy among CYP2C19 genotypes

| Authors | Number of patients | Events | Diarrhea/loose stools | Dysgeusia | Abdominal pain |
| --- | --- | --- | --- | --- | --- |
| Dojo et al. (18) | 170 | 15 | 12 | 3 | 0 |
| Inaba et al. (1) | 183 | 53 | 39 | 1 | 1 |
| Kawabata et al. (2) | 187 | 108 | 65 | 24 | 24 |
| Miki et al. (3) | 145 | NA |  |  |  |
| Take et al. (4) | 249 | 51 | 40 | 0 | 0 |
| Isomoto et al. (5) | 61 | 7 | 5 | 0 | 0 |
| Sheu et al. (6) | 200 | 54 | 11 | 10 | 11 |
| Okudaira et al. (19) | 89 | NA |  |  |  |
| Kuwayama et al. (7) | 225 | 119 | 87 | 46 | 0 |
| Higuchi et al. (8) | 288 | UN | 82 | 0 | 0 |
| Furuta et al. (9) | 150 | NA |  |  |  |
| Kuwayama et al. (10) | 479 | 215 | 134 | 25 | 11 |
| Hagiwara et al. (11) | 22 | 3 | 1 | 1 | 3 |
| Lee et al. (20) | 492 | NA |  |  |  |
| Zhang et al. (21) | 240 | 15 | 2 | 0 | 1 |
| Prasertpetmanee et al. (22) | 110 | 33 | 0 | 10 | 0 |
| Yang et al. (12) | 150 | 53 | 12 | 15 | 0 |
| Liou et al. (13) | 650 | 271 | 93 | 92 | 32 |
| Phiphatpatthamaamphan et al. (14) | 50 | UN | 6 | 18 | 8 |
| Chunlertlith et al. (15) | 170 | UN | 3 | 93 | 0 |
| Shimoyama et al. (23) | 200 | NA |  |  |  |
| Ozaki et al. (16) | 147 | NA |  |  |  |
| Arévalo Galvis et al. (24) | 69 | 42 | 6 | 12 | 0 |
| Chen et al. (17) | 338 | 124 | 53 | 50 | 28 |
| Lee et al. (25) | 254 | 12 | 1 | 4 | 1 |

Abbreviations: NA, not available; UA, unknown
